# ANOVA-Like Differential Expression (ALDEx) Analysis for Mixed Population RNA-Seq

**DOI:** 10.1371/journal.pone.0067019

**Published:** 2013-07-02

**Authors:** Andrew D. Fernandes, Jean M. Macklaim, Thomas G. Linn, Gregor Reid, Gregory B. Gloor

**Affiliations:** 1 YouKaryote Genomics, London, Ontario, Canada; 2 Department of Biochemistry, The University of Western Ontario, London, Ontario, Canada; 3 Department of Microbiology & Immunology, The University of Western Ontario, London, Ontario, Canada; 4 Department of Surgery, The University of Western Ontario, London, Ontario, Canada; 5 Canadian Research & Development Centre for Probiotics, Lawson Health Research Institute, London, Ontario, Canada; Hospital for Sick Children, Canada

## Abstract

Experimental variance is a major challenge when dealing with high-throughput sequencing data. This variance has several sources: sampling replication, technical replication, variability *within* biological conditions, and variability *between* biological conditions. The high per-sample cost of RNA-Seq often precludes the large number of experiments needed to partition observed variance into these categories as per standard ANOVA models. We show that the partitioning of within-condition to between-condition variation cannot reasonably be ignored, whether in single-organism RNA-Seq or in Meta-RNA-Seq experiments, and further find that commonly-used RNA-Seq analysis tools, as described in the literature, do not enforce the constraint that the sum of relative expression levels must be one, and thus report expression levels that are systematically distorted. These two factors lead to misleading inferences if not properly accommodated. As it is usually only the biological between-condition and within-condition differences that are of interest, we developed ALDEx, an ANOVA-like differential expression procedure, to identify genes with greater between- to within-condition differences. We show that the presence of differential expression and the magnitude of these comparative differences can be reasonably estimated with even very small sample sizes.

## Introduction

RNA-Seq has been widely adopted by the biomedical research community and used to interrogate gene expression in many organisms. The potential advantages of RNA-Seq as a gene-expression profiling method are indisputable[1]. RNA-Seq, in principle, can be used even when the genome sequence of the organism is not available because the sequence of each transcript itself can be identified and compared to closely related organisms (e.g. [2]). In some cases *de novo* assembly is even possible[3]. RNA-Seq can be used to discover unanticipated transcripts, to identify and characterize novel splice and promoter variants[4]-[6], and has a much larger potential dynamic range than microarrays[7]. There are fewer false positive transcripts identified with RNA-Seq than with microarrays[8]. The underlying sequencing platforms display excellent within- and between-platform reproducibilities[7], [9], without the *ad hoc* corrections common with microarrays[10].

One expected outcome from an RNA-Seq experiment is a list of genes for which there is evidence of differential expression between two experimental conditions. A large number of tools have been developed to infer such a gene list, and these tools have evolved as researchers have used increasingly sophisticated statistical methods to estimate and compare relative transcript abundances from sequencing read counts[11].

Existing RNA-seq analysis tools, reviewed in Pachter[11], were designed to examine datasets derived from single organisms. These tools use a fixed-effect analysis to infer differential expression, where the observed gene expression level is equal to the expected expression level for that sample's experimental condition plus some general, random error. The single error term accounts for variation due to three sources: sampling and technical replication, as well as per-sample variability. Implicit in fixed-effect models is the assumption that within-condition differences are not of direct interest; expression level variation *within* an experimental condition is assumed to be either negligible compared-to or simply considered part-of the overall error. However, Meta-RNA-Seq involves changes in both organism abundance and transcript abundance, and these can be confounded using existing analysis tools.

The main purpose of this work is to show that within-condition variation *cannot* reasonably be ignored, especially in Meta-RNA-Seq experiments, and if such variation is not correctly accommodated for then misleading inference will occur. We show that single organism RNA-Seq and Meta-RNA-Seq datasets can be examined by robust statistical methods similar to traditional random-effect ANOVA models that decompose sample-to-sample variation into four parts: within-condition variation, between-condition variation, sampling variation, and general (unexplained) error. We propose that evidence for differential expression in these datasets can be accurately evaluated using both ''statistical significance'' and ''effect-size'' estimates derived from such models because these two values respectively describe the confidence we have that expression in the conditions are different, and the magnitude by which they differ. Others have argued that characterizing biological data in this way is more informative than decisions based upon *p*-value thresholds because *p*-values encourage acceptance or rejection of a null hypothesis rather than an explicit assessment of the evidence[12], [13]. We further model the data as ''compositional'' or ''proportional'' because the reads obtained on a high-throughput sequencing run are constrained to the total number of reads available, the total of which are, to a large extent, non-informative. Spurious correlations are observed when compositional data are analyzed[14] and recent methods to deal properly with high-dimensional proportional data have been developed[15]–[18].

## Results

Most RNA-seq datasets contain many genes with zero read counts (e.g. [2], [19]–[21]) due to sparse sampling. The method described below explicitly accounts for the probability that genes with 0 read counts actually represent non-expressed genes as opposed to insufficient sequencing depth.

Most statistical analyses of RNA-Seq data model the read counts obtained from the sequencer as having come from a Poisson-like process such that for gene 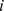 the number of counts observed 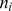 is a Poisson random variable from a process with rate 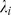. These analyses seek to infer 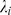 given 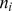 for every gene in the sample. For a Poisson random variable the mean and variance equal both the rate, i.e., 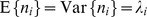. However, plots of 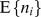 versus 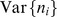, where each quantity is estimated through technical replicates, have been used to argue that 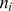 is over-dispersed. Thus when 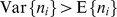, such over-dispersion implies that the 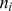 are better modelled by a negative-binomial-like process [22]. As shown below the over-dispersion attributed to sampling variance is equally and independently well-explained by putative technical error or within-condition variance. In fact, the original observation of overdispersion was from the analysis of SAGE data [22] where the additional dispersion parameter was added to account for library-to-library variability. Note that the term ''technical replicate'' is imprecise as it may refer to error introduced by the sequencing technology or error introduced during sample preparation [19], [23]. In this work, ''technical replicate'' is used in the sense of Robinson and Smyth [22] that includes error due to sample preparation.

### Inferring proportions from counts

The total number of reads 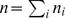 observed from a single high-throughput sequencing run, although itself a random variable, provides little direct information about the sample and is generally not of interest. Instead, for each gene 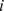 we use the set of counts 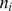 to infer its proportion 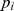 within the sequenced sample. We do so by assuming that each gene's read count was sampled from a Poisson process, i.e., 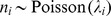 with 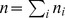. The equivalency between Poisson and multinomial processes can then be used to assert that the set of joint counts with given total has a multinomial distribution, i.e., 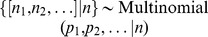 where each 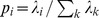.

Unlike previous methods, however, we exploit the fact that *formal* equivalence between Poisson and multinomial processes does not imply *inferential* equivalence. Specifically, rather than using 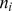 to estimate 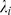 and then using the set of 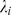 to estimate 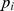, we estimate the set of proportions 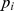 directly from the set of counts 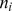. Critically, an often-overlooked fact with such estimates is that the traditional maximum-likelihood estimate of 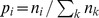 for multinomial processes is accurate only when none of the counts 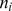 are small or zero [24]. Since most datasets of this type contain large numbers of genes with zero or small read counts, the maximum-likelihood estimate of 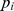 is often exponentially inaccurate. For example, if a coin is flipped twice and comes up heads both times, the heads-to-tails counts 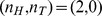 do not imply that the probability of tails 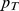 is exactly zero. In fact, assuming that 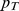 is exactly zero is equivalent to the rather strong assumption that the coin has only one side. Equivalent statements hold for sequencing counts; the assumption that a read count of zero is equivalent to the transcript being ''not present'' has surprisingly large consequences on the overall analysis, as we show later.

Instead of maximum-likelihood, we use standard Bayesian techniques[25] to infer the posterior distribution of 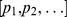 as the product of the multinomial likelihood with a 
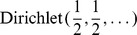
 prior. Note that by definition and construction, Dirichlet-distributed random variates always enforce conservation of proportion such that 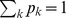. For an overview of Dirichlet random variables, see Frigyik *et al* [26]. Further, this specific choice of prior and this choice alone has been shown to simultaneously maximize the information present in the data while minimizing the influence of the prior on the posterior when the relative frequencies of all genes are of equal interest[27], as is the case for RNA-Seq or other tag-sequencing type experiments. This is the only method of inferring frequencies from counts that is guaranteed to be invariant to reparameterization, have consistent sampling properties, and be immune to marginalization paradoxes [28], [29].

Due to the large variance and extreme non-normality of the marginal distributions 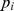 when the associated 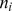 are small, we do not summarize the posterior of 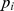 using point-estimates. Instead, all inferences are performed using the *full* posterior distribution of probabilities drawn from the Dirichlet distribution such that 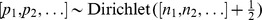.

This multivariate distribution ensures that none of the inferred proportions are ever exactly zero even if the associated count is zero, and that probability is conserved (i.e., 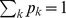). The given posterior further explicitly accounts for the fact that a read count of 1 out of 100 total reads conveys much lower precision of an estimated proportion than does a read count of 100 out of 10000 total reads, even though they have the same fractional read count. Therefore, marginal distributions of 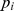 are wide when the associated read count is low and narrow when the associated read count is large, as would be intuitively expected.

In other words, rather than transforming the observed data through an *ad hoc* normalization procedure and then inferring proportional expression, we instead prefer to infer proportional expression *directly* from the read count observations through a statistical model that, by construction, explicitly always enforces ''normalization'' of the inferred parameters.

### Transforming proportional data into independent components

It can be difficult to meaningfully compare between-sample values from proportional distributions because each set of proportions is constrained to have a constant sum. This means that the values cannot be independent; an increase in one or more proportions *necessarily* implies a concomitant decrease in one or more other proportions and *vice versa* [14]. Fortunately, Aitchison and Egozcue, among others[15], [17], [18] have developed procedures to transform component proportions into linearly independent components. The transformation can be understood by considering a hypothetical two-gene experiment where genes *H* and *T* are inferred to have proportional expression 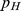 and 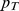, respectively and that 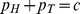. The sum, 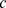, is a positive constant scaling factor that is usually equal to one (proportional data) or one hundred (percentile data). The proportional two-component vector 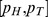 is experimentally equivalent to 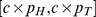 for any positive scale-factor 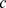. Taking component-wise logarithms, we see that 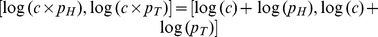, which, after algebraic rearrangement can be written as 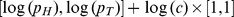. Egozcue *et al.* showed that this space of log-proportions was equivalent to a Euclidean vector space[17], [18] and as such, contributions from the subspace spanning 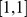, here termed the ''uninformative subspace,'' can be removed via standard techniques from linear algebra. More explicitly, for two or more dimensions the overall procedure for a set of 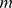 proportions 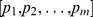 involves taking component-wise logarithms and subtracting the constant 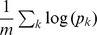 from each log-proportion component. This results in the values 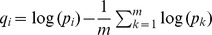 where 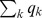 is always zero, and this transformation from 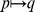 has been named the Isometric Log-Ratio (ILR) transformation[18]. Most importantly, projecting 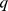 onto any basis of its 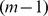-dimensional span results in a vector with linearly independent components. For 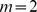 the ILR transformation is shown graphically in Supplementary Figure S1 in File S1.

### Properties of the transformed data

Removing the uninformative 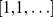 subspace has the critical effect of removing the possibly-large multivariate statistical bias introduced by the log-transformation (see Supplementary Figure S1 in File S1). Furthermore, although the constraint that 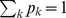 induces unavoidable covariation among genes, the covariance induced in the adjusted log-proportions 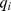 can be shown to be inversely proportional to the number of genes considered, as shown by the explicit formula for 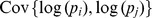 given below. For high-throughput data where thousands of genes are simultaneously considered, this induced covariance becomes effectively zero. Although still not independent since 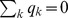, carefully-constructed analyses can exploit this near-zero covariance in order to simplify numerical computations.

The adjusted log-proportion values 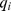 correspond exactly to ''fold-based'' abundances from traditional expression analysis that have been shifted so that the mean log-expression value is zero. The result is that the values of 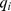 can be any real value centred on zero. Using base-two logarithms, for example, makes 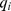 represent the two-fold doubling or halving of proportional level. Next, since each 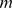-dimensional vector 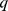 can be exactly represented by a 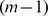-dimensional vector 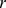 whose components are linearly independent, 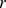 and hence 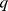 can be analyzed in a traditional ANOVA-like framework. We emphasize that although the analytic distribution of 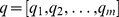 is straightforward to compute given the Dirichlet-distributed 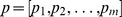, it is cumbersome to use directly. Thus we estimate the distribution of 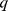 from multiple Monte Carlo realizations of 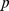 given 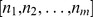. When summary statistics are necessary, however, we note that for 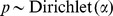 we have 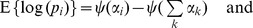


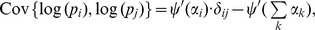
where 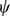, 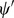, and 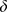 represent the digamma, trigamma, and Kronecker-delta functions, respectively. These formulas are derived using the standard exponential family formula for the moment generating function of the sufficient statistic 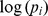 for the Dirichlet distribution. Note that since each 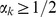 and the trigamma function 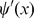 is roughly proportional to 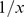, the 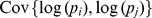 matrix becomes diagonal at a rate inversely-proportional to both the total read count and the number 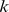 of genes considered, when 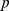 is Dirichelt-distributed as described.

### Application of the method to real datasets

Table 1 shows the characteristics of three distinct RNA-seq datasets used in the analysis. Each dataset contains four samples, two in each condition. The first dataset is an RNA-Seq experiment on the Illumina platform performed by Marioni et al[19] that contains technical replicate samples of gene expression in Liver and Kidney cells, where the error due to sample preparation is known *a priori* to be zero. We refer to this to as the ''L-K dataset'' and we used replicates 1 and 2 of Kidney and of Liver run in lanes 1 and 2. This dataset is often used to evaluate assumptions underlying RNA-Seq methods[23], [30] and contains 32000 coding sequences (CDS). The second dataset is a very high sampling depth RNA-Seq experiment performed by our group on the ABI-SOLiD 4 platform to examine differential expression upon shift from growth at pH∶7.2 to 5.5 in *Bacillus cereus* 14579. One of the two samples in each growth condition contained a plasmid. Transcripts from the genes on the plasmid may be differential because of differential presence, differential expression, or both, and some chromosomal genes have differing expression because of the presence or absence of the plasmid. For simplicity, we will refer to these as plasmid-associated genes. In this data set, technical replicates *included* error due to sample preparation. The third dataset is a meta-RNA-Seq experiment performed to characterize the differences in gene abundance and expression in the vaginal bacterial communities found in two women with a micobiota associated with vaginal health and two women with a microbiota characteristic of a dysbiotic state called bacterial vaginosis[31]. Table 2 shows the organism composition of these four samples. We will refer to this as the Meta dataset.

**Table 1:** Sample characteristics

Sample	CDS	Reads/sample	0 read genes/sample
L–K	32000	1.43–1.98 M	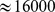
*B. cereus*	5378	16.7–23 M	50–63
Meta	33412	5.5–10.6 M	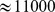 – 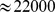

Sample names, coding sequence (CDS) numbers, the range in mappable reads per sample, and the number of genes with 0 reads in any sample are given.

**Table 2:** Meta sample taxonomic abundance

Taxon	N4	N30	B27	B31
*Gardnerella vaginalis*	0.0	0.0	21.3	5.5
*Lactobacillus*	7.6	7.6	0.0	2.4
*Lactobacillus acidophilus*	1.5	1.6	0.0	0.4
*Lactobacillus crispatus*	86.0	79.7	0.2	23.4
*Lactobacillus iners*	1.1	7.0	25.8	53.4
*Lactobacillus jensenii*	2.9	3.2	0.0	1.2
*Megasphaera* sp.	0.0	0.0	9.1	0.3
*Prevotella amnii*	0.0	0.0	40.5	6.9
*Prevotella disiens*	0.0	0.0	0.1	1.2
*Prevotella timonensis*	0.0	0.0	0.3	2.0
rare	0.8	0.8	2.5	3.3

The organism name and proportional abundance for the two clinical samples with normal Nugent scores (N) and the two samples with high Nugent scores indicitive of bacterial vaginosis (BV) are given. Totals may not sum to 1 because of rounding errors.

For each sample discussed below, sequencing reads were mapped to genes and hence gene transcripts using standard techniques[32] resulting in a set of read counts for every sample. The count tables for the L-K dataset were accessed from the supplementary data of Marioni et. al.[19]. For each sample, the set of read counts 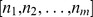 was used to infer a posterior distribution for the relative abundances 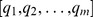, as outlined above.

**Figure 1:**
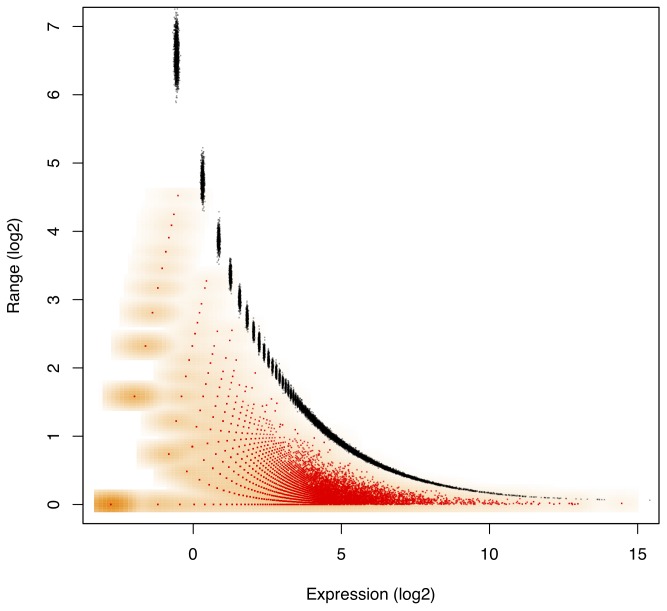
Dirichlet-distributed proportions accurately account for the sampling variance. This plot overlays the expected range between the 1–99% quantiles for 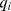 with observed range of 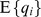 computed for the Liver library replicates in the L–K dataset. Marioni et al[19] minimized technical error with an experimental design where the same Illumina library was run in two separate lanes. Monte Carlo Estimates of 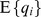 are shown in red with the density of the values shown in orange, while 1–99% expected quantile ranges from the Dirichlet are shown in black. This demonstrates that the error inherent in high-throughput sequencing is greatest when the counts are small and least when the counts are large. The near-perfect overlay of actual and modelled values strongly support idea that modelling proportions through a Dirichlet-multinomial process accurately accounts for the sampling variance inherent in RNA-Seq, and by extension in other high-throughput sequencing analyses. The error in estimating the expected quantiles is observable by the size of the points plotted in black and becomes small when expression is non-trivial. Values on the 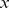-axis were calculated with the given formula for 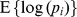 and were adjusted to remove the non-informative subspace as outlined in the text. Thus the 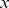-axis value of zero corresponds to the expected per-gene log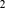-expression value.

Figure 1 shows a plot of the dispersion caused by sampling variation for the L-K dataset. Marioni et. al.[19] assessed sampling variability by running the same library on separate Illumina lanes. As predicted, observed variability is large when a gene is covered by zero or few reads and small when a gene is covered by many reads. To assess the correspondence between the actual variability and the variability modelled by the Dirichlet distribution, the expected difference between the 1–99% quantiles for 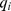 are overlaid with Monte Carlo estimates of 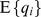 computed from realizations of the posterior distribution of 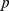. The almost perfect overlay of these values strongly supports the idea that modelling proportions through a Dirichlet-multinomial process accurately accounts for the sampling variance inherent in RNA-Seq, and by extension in other high-throughput sequencing analyses, when technical or other errors are not predominant. Others have also suggested that this distribution is appropriate for these types of data[33]–[35].

**Figure 2:**
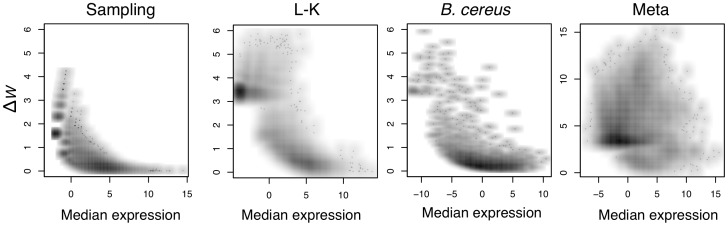
Sampling and technical variance is distinct from within-condition variance. A comparison of within-condition gene expression-difference to the median expression level for three different experiments. The left panel shows the sampling variance for comparison and the three experiments are shown in subsequent panels. The L-K RNA-Seq data set compares gene expression in two liver and two kidney samples. The *Bacillus cereus* RNA-Seq data set for samples grown at neutral pH and two samples 20 minutes after shift to grown at low pH. Meta-RNA-Seq data is for microbial gene expression analysis of four clinical vaginal samples from two women with a healthy microbiota and two women with a microbiota indicative of bacterial vaginosis. The RNA-Seq experiments in the L-K and *B. cereus* datasets were from controlled conditions with identical gene content per condition and show that the vast majority of highly-expressed genes have small within-condition estimates of 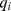, and that estimate only becomes imprecise as 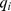 becomes very small. The Meta-RNA-Seq panel shows that when within-condition variance is high, there is no relationship between the expression level and the within-condition variance. Note that base-2 logarithms were used throughout.

The importance of distinguishing sampling and technical variance from within-condition variance is illustrated by Figure 2 which shows the maximum within-condition expression difference versus the median expression for the sampling replicate experiment described above and for the three datasets in Table 1. The L-K and *B. cereus* RNA-Seq experiments were tightly controlled and much of the observed variance can be attributed *a priori* to sampling error. We observe that the majority of genes with high expression had their proportions estimated with high precision, and this precision was directly proportional to the expression level. The fourth panel of Figure 2 shows a corresponding variance-*vs.*-median plot for the Meta-RNA-Seq analysis of the vaginal meta-transcriptome. Here, we see that biological variability is independent of expression level and much larger than can be explained by sampling variability alone. Thus, for the Meta-RNA-Seq data much higher variance due to biological variability is observed since organism abundances and the underlying gene abundances can vary in addition to transcript levels when samples contain multiple organisms.

### Identifying differentially expressed genes

Pachter[11] has pointed out that the majority of existing RNA-Seq statistical analysis tools can be distilled down to one of a few basic methods, and all are expected to converge on the same result with asymptotically large data sets. Informally speaking, existing methods are essentially equivalent to the following fixed-effect ANOVA. Given gene 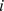 in condition 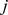 with replicate number 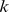, they model



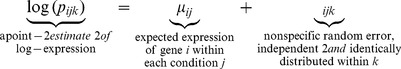
and test the hypotheses that



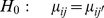
between conditions 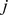 and 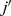, for all genes 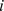. As is usual in discussions of ANOVA, 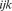 is assumed to be approximately Normal. It is important to realize that under this model, within-condition sample-to-sample variation is assumed to be small and essentially negligible compared to 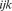. It is specifically this assumption that we believe to be generally untrue for RNA-Seq analyses.

In contrast, our model is fundamentally different from currently available general-purpose metagenomics analysis packages in its assumption that within-condition expression is itself a non-negligible random variable. For example, two metatranscriptomic samples taken from within the healthy condition may have substantially different gene expression due to the different microbial populations present in each sample. Random-effect ANOVA models specifically account for this type of within-condition variance and the one employed herein, informally speaking, models



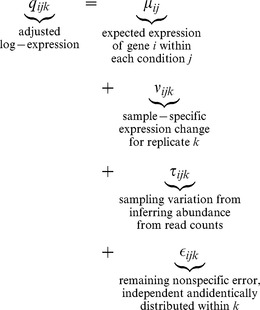



Again as per the usual ANOVA assumptions, 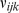 is assumed to be approximately Normal. The distribution of the sampling error 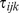 is given by the adjusted log-marginal distributions of the Dirichlet posterior. As seen through empirical observation, 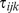 is very Gaussian-like and sharply defined when its associated read counts are large and becomes progressively more diffuse and non-Normal as read counts drop. This empirical observation matches expected behavior since, prior to removal of the uninformative subspace, each 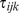 is the beta-distributed marginal of a Dirichlet distribution. The sample-specific expression 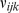 represents the random differences between replicates 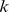 and 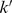 for gene 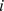 in condition 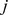 and can specifically account for factors such as technical differences or differing population structures. Subject to standard ANOVA identifiability constraints, we can also test the hypotheses 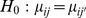. Although this hypothesis test can convey statistical significance, it does not imply that the conditions 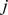 and 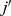 are meaningfully different. Instead, such meaning can be inferred through an estimated effect-size that compares predicted between-condition differences to within-condition differences.

There is a vast literature describing the analysis of classic random-effect ANOVAs, but these are generally inapplicable to RNA-Seq data for three reasons. First, the extreme non-normality induced by genes with low read counts invalidates standard techniques such as those using 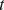-tests or 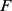-tests. Second, there are almost always too few samples to properly support or refute the ''equal variance'' postulates necessary to most ANOVA setups because it is still cost-prohibitive for most labs to sequence more than a few samples per condition. Finally, the constraint 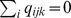 implies that estimates derived from component 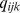 values cannot be made independently among genes 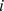. These reasons result in most RNA-Seq experiments leaving too few degrees of freedom to properly estimate all parameters even if normality of 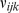 is assumed; itself a rather strong assumption.

It is worth noting that Blekhman *et al.* [36] used a similar model for the analysis of sex-specific and lineage-specific alternative splicing in primates, where the Poisson intensities were modelled as fixed effects with a random-effect error term. Our work differs from theirs in two important aspects, however. First, rather than normalizing intensities, treating them as point-estimates from the read counts, we effectively integrate over all intensities consistent with the observed counts. This is important when dealing with samples that have many genes and hence relatively few reads per gene. Second, our method is optimized to allow reasonable inference even when only two samples are given in each group. Rigourous statistical inferences are difficult to make in such situations as, depending on prior assumptions, the posterior distributions of various statistics are often vary heavy tailed.

To ameliorate these difficulties, we have developed an ''approximate ANOVA''-like procedure suitable for the analysis of small-replicate experiments such as the majority currently described in the literature. Robust estimators, i.e., medians rather than means, are used throughout in order to mitigate effects of heavy tails and skewness prevalent in low read count genes. In what follows, let 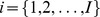 index genes, 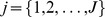 index the condition, and let 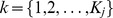 index the replicate of a given condition. Recall that the set of 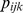 are random variables since they represent the posterior distribution of parameter estimates. Similarly, the set of 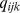 are random variables as they are simple functions of the 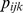. Now consider the following random variable mixtures.

- The within-condition mixture 
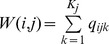

- The absolute fold difference between-conditions 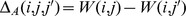

- The between sample, within-condition difference 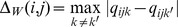

- The relative effect-size 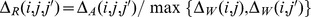

We emphasize that these quantities are random variables, not traditional point-estimates, and thus have distributions that can be trivially estimated via standard Monte Carlo realization. The distribution of 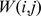 is termed the ''within-condition distribution'' of gene 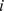 within condition 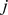, and the distribution of 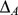 is termed the ''between-condition'' distribution. Note that the ''max'' operator in the definition of 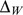 makes it a conservative surrogate for the pooled within-condition variance across all conditions. Since the distributions of 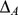, 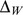, and 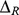 are estimated from multiple independent Monte Carlo realizations of their underlying Dirichlet-distributed proportions for all genes 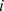 simultaneously, they intrinsically obey the requisite 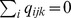 constraint and are thus invariant to differing total read counts. Note that although the denominator of 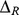 can be zero, this occurs with probability zero and 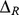 remains well defined, much as the Cauchy random variable can be described as the ratio of two Gaussian random variables.

Due to the small number of experimental replicates often used, the distributions of these random variables can be fairly broad or possess heavy tails. We therefore summarize them via their quantiles, using the notation of 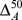, and 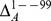 to denote the median, and 1-99% quantiles for 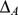, respectively, and so on for the others. We further identify the quantile of zero in the distribution of 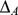 as 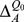. Note we use a symmetric variant of 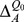 such that 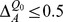. Importantly, we do not replace the estimators with their point-summaries too early, since for 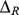 the median of a ratio can be quite different than the ratio of medians.

**Figure 3:**
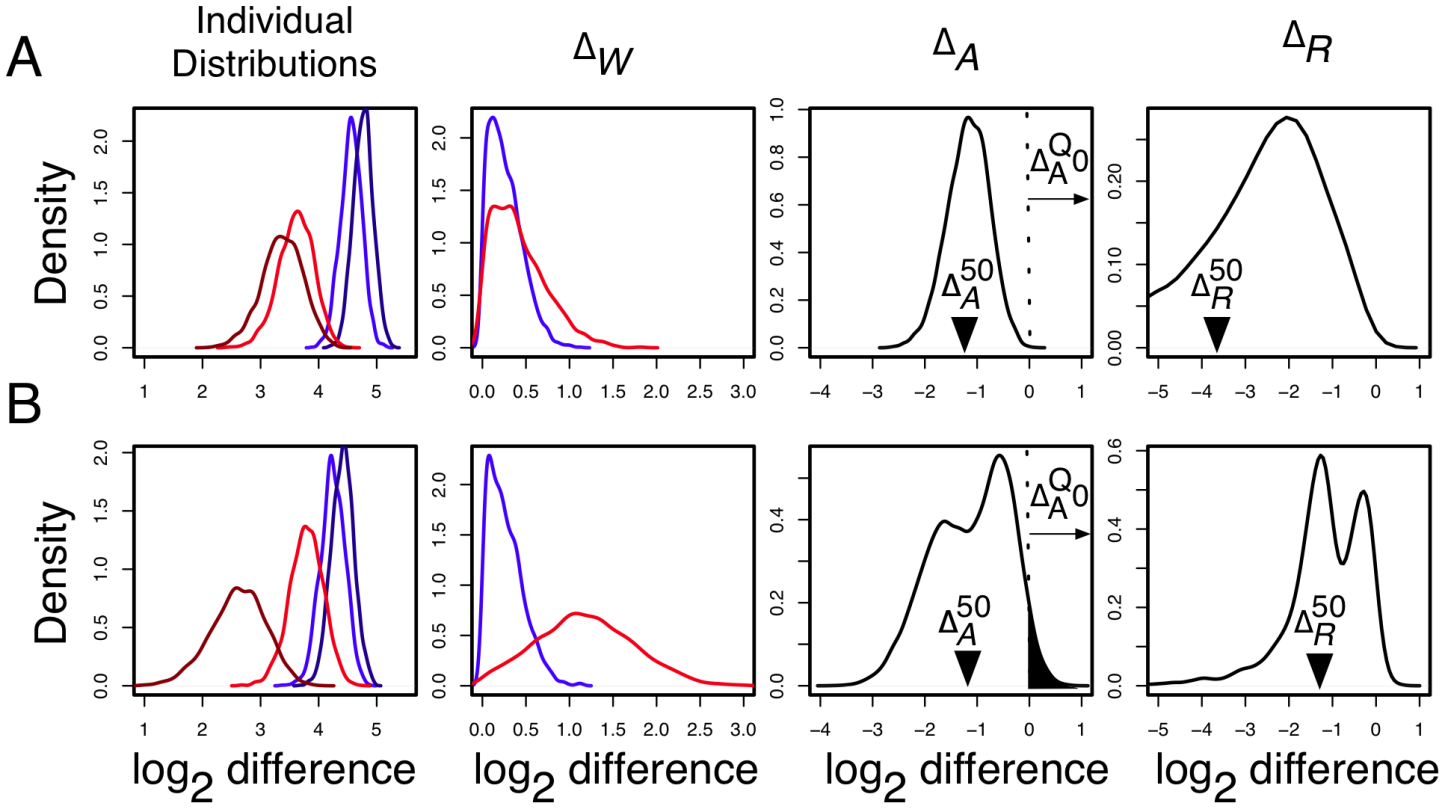
Approximate ANOVA via Absolute and Relative fold differences. The figure shows how the method explicitly accounts for the within-condition dispersion using as an example two genes with similar absolute fold differences (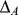) of -1.17 and -1.13 but very different relative fold differences (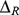) and 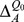 values in the L-K dataset. Dirichlet sampled distributions are generated from the raw read counts as described in the text. These distributions are log-transformed and the noninformative subspace is removed. Posterior distributions of 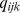 are shown for 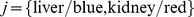 and for replicates 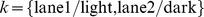. Both genes are abundantly expressed in this dataset, with median expression levels between 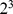 and 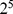 greater than the mean across-gene expression level. 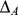 is computed by randomly sampling one of the red distributions, randomly sampling one of the blue distributions, and subtracting for all pairs of between-condition distributions. 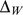 is computed by sampling a light and a dark from each red and blue distributions, subtracting light and dark, and selecting the difference with the greatest magnitude. 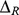 is computed as the ratio of a single realization of 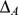 and 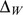, and is computed for each realization of 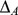 and 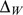. The 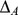 distribution is narrower in A than in B implying a greater precision in estimating this value. This precision can be estimated by 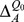, the quantile of zero in 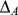, which is shown graphically as the black-filled area under the 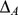 distribution curves. The 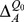 values are 0.0001 and 0.035 in panels A and B respectively. The vertical arrows show the median values of 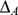 and 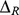. Thus, the between-condition expression values for the gene in Panel A are scored as separable by ALDEx (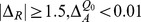) but not for the gene in Panel B (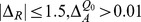). These conclusions agree with inspection-based intuition from examining the initial adjusted log-expression distributions that are shown in the left panel.

A graphical depiction of our approximate-ANOVA is depicted in Figure 3. It begins by computing multiple Monte Carlo realizations of 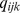 via Dirichlet samples 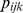 based on each sample's read count. Realizations are grouped (as if they were drawn from a mixture-distribution) across replicates and between-conditions to compute realizations of 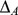. Grouping within-conditions and between samples yields realizations of 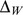. The ratio of these two realizations yields a single realization of 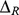.

**Figure 4:**
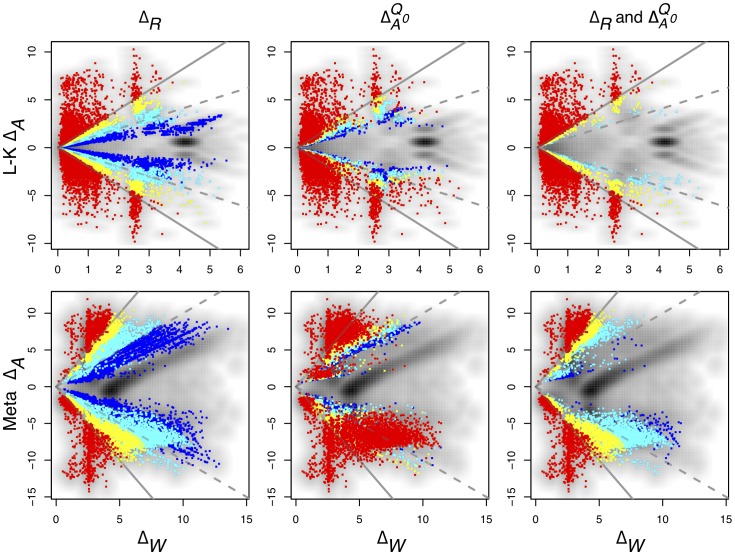
Fold-change to variance (MW) plots of different ALDEx cutoff values. Plotted here are 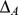 (i.e., the 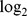-fold expression changes) vs. 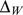 (i.e., the maximum within-condition expression differences) for the L-K and Meta-RNA-Seq datasets. The grey background shows the density plot of the values. By construction, threshold 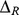 values of 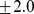 should select genes for which the between-condition variation is reasonably likely to be at least twice the within-condition variation. This threshold is illustrated by the solid grey line, and the within/between-condition equivalence line is shown as the dashed grey line. The effect of altering the 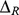 and 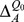 cutoffs is shown. Large 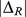 values identify genes with a larger between-condition differential expression than within-condition variation. The 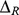 values were 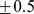 (blue), 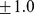 (cyan), 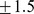 (yellow) and 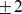 (red). There are a number of points where the 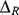 values exceed the chosen threshold in both the L-K and Meta datasets. The 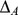 column shows the effect of identifying genes based on the estimated proportion of 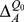. These are plotted for values of 0.1, 0.05, 0.01 and 0.001 in the two datasets, again coloured as blue, cyan, yellow or red respectively. Here we observed that the 0.01 and 0.001 cutoffs were very similar and the larger cutoffs admitted many more genes with high within-condition variation. The third panel shows the effect of enforcing the 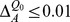 along with each of the 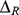 cutoff values. In this case we observe that a 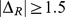 combined with an 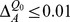 is sufficient to ensure that genes identified as differentially expressed always have lower 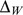 than 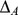 values in both datasets. Using both values in combination allows arbitrarily small fold expression differences to be identified if they are supported by high within-condition concordance.

Being an estimated effect-size, 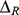 can be used to identify genes where expected between-condition differential expression is likely to be meaningfully larger than expected within-condition differential expression. The ability of different 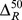 values to distinguish those genes that have larger between-condition than within-condition variance is shown in Figure 4. As a theoretical minimum, the criterion that 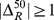 can be used to select genes where expected between-condition changes are of the same order or greater than the expected within-condition changes. However, this figure shows that 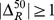 is too inclusive.

For genes with low read counts, the 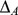 and 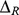 distributions can be overly-broad with long tails. Thus we introduce a further criterion that is loosely analogous to a fixed-effect ANOVA 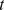-test. Supplementary Figure S2 in File S1 shows that enforcing the criterion whereby 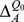 is small selects against genes with low read counts, and the *within* and *between* condition variation characteristics of genes selected by different values of 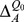 are shown in Figure 4. The results of using the combined 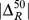 and 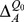 criteria are described by the third panel of Figure 4. In practice, we find that 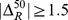 and 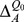 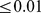 is a minimum practical threshold that avoids an overabundance of false-positive identifications regardless of the dataset, and recommend 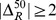 for use when a conservative estimate is required. We emphasize that, especially for experiments with small replicate numbers, it is critical to examine the suggested criteria to ensure there is a match between the characteristics of the data being examined and the threshold values chosen. This will ensure that the decision as to which genes are identified as differentially expressed is based on analyses similar to the plot in Figure 4. Changing threshold values corresponds to adjusting the stringency of a hypothesis test, with concomitant trade-off in resultant Type-I and Type-II errors. However, as shown in Figure 4 the cutoffs chosen ensure that no genes with large within-condition variance relative to between-condition variance are identified as differentially-expressed in any of the datasets tested.

### Inputs and Outputs

**Figure 5:**
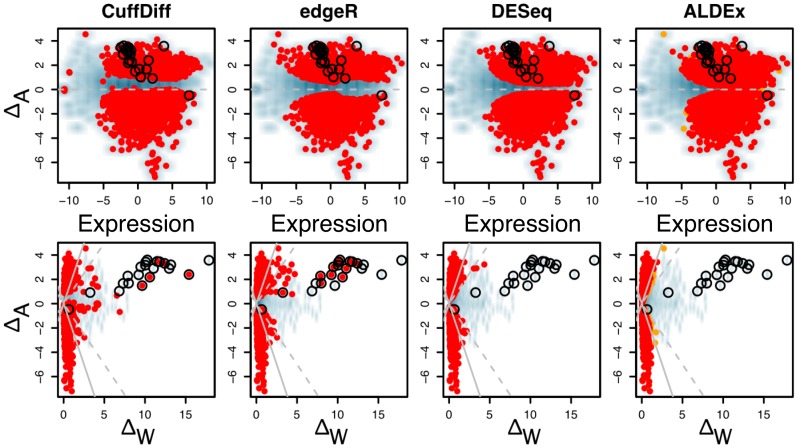
Comparison of four differential expression methods in the *B. cereus* dataset. Transcript abundances identified as differential by the first three methods are highlighted in red on a background density plot. Default false discovery rates for each program were used, 0.05 for CuffDiff and 0.1 for both edgeR and DESeq, since these reflect the configurations in which most users will use these programs. In the case of ALDEx transcripts with 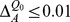 are highlighted in red and orange for 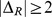 and 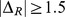. Transcripts originating from genes contained on the plasmid that is found in one sample from each condition are circled. The top row shows typical Bland-Altman style (MA) plots where the median absolute fold change (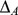) is plotted vs. the mean expression value (Expression). The mean expression value on x-axis is 0 for the reasons outlined in the text. Notice that the edgeR method identifies differentially-expressed transcripts with much lower abundances than the other three methods. The plasmid-encoded genes are not differentiated on the Bland-Altman-style plots. The bottom row shows an MW plot of the median absolute fold change between-conditions (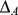) vs. the maximum within-condition difference (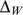) of the same data. Here it is clear that transcripts originating from the plasmid-encoded genes exhibit very large 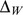 values. Interestingly, there are a number of chromosomally-encoded genes in this dataset, and in the other two (see previous figure) that also show large 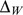 values, demonstrating that within-condition variation can be problematic even for samples derived from well-controlled conditions. Both CuffDiff and edgeR identified as differentially expressed a significant fraction of the plasmid-derived transcripts.

The method has been implemented as the ALDEx (ANOVA-Like Differential Expression) version 1.0 package for the R statistical software system. The ALDEx package takes as input a table containing per-gene sequencing counts for each replicate and a list indicating which replicate should be grouped in what condition. Each sample is a column, and per-gene counts are in rows. It currently requires two or more samples in two conditions. It computes one table for all genes containing the following: individual median expression data for each gene in each sample, the median expression for each gene in each condition, the median expression level across conditions (A), the median absolute fold difference (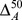), the median effect-size (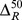), the median within-condition difference (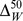) and the quantile of zero in 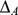 (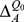). Optionally, multiple quantiles in addition to the median are also returned. Graphically, these data may be displayed as Bland-Altman (MA)[37] and MW plots similar to those in Figure 5 and Supplementary Figure S2 in File S1.

### Comparison of ALDEx to existing methods

We initially compared the ability of ALDEx, DESeq[23], edgeR [38] and CuffDiff[39] to identify differentially expressed transcripts in the *B. cereus* dataset. Gene expression was evaluated for *B. cereus* ATCC 14579 grown *in vitro*. This dataset contained four samples in two conditions, two samples before and two samples 30 minutes after a pH∶7.2 to pH∶5.5 shift. Importantly, only one sample per condition contained the plasmid pBClin15. In this experimental design chromosomal genes exhibited high between-condition expression differences while, the genes on the plasmid and those controlled by genes on the plasmid exhibited both high within-condition variability and high between-condition expression differences.

**Figure 6:**
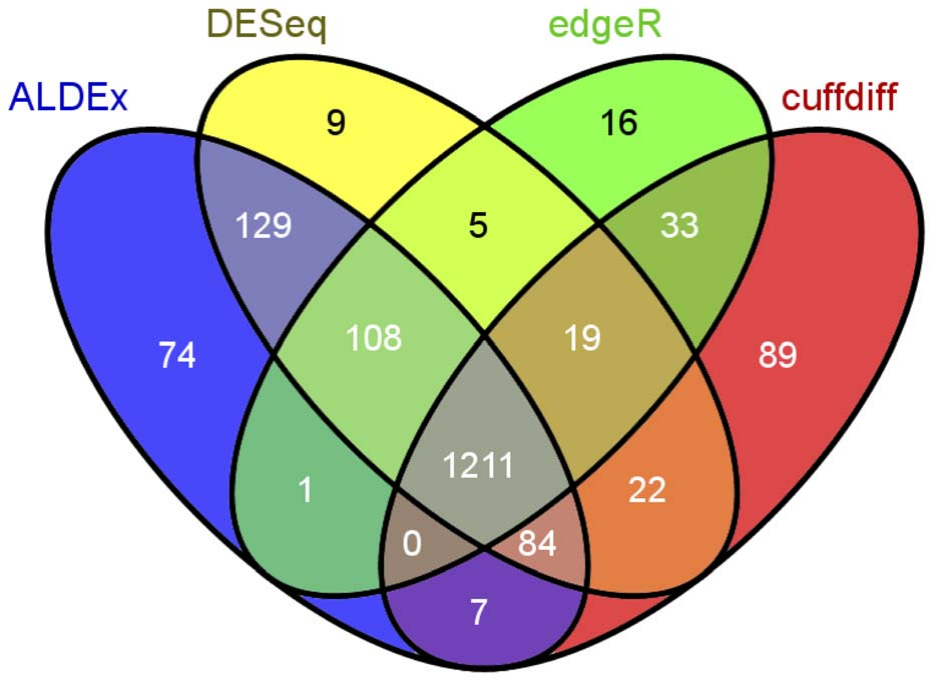
Venn diagram of the four differential expression methods in the *B. cereus* dataset. Transcript abundances were identified as differential as in Figure 5. The overlap between the number of differentially expressed transcripts for each method is given in the individual cells of the diagram. The number of differentially regulated transcripts for each method is: ALDEx 1614, DESeq 1587, edgeR 1393, CuffDiff 1465. The diagram was prepared using the Venny web tool(Available: http://bioinfogp.cnb.csic.es/tools/venny. Accessed May 23, 2013) [57].

The results are shown in Figures 5 and 6. The between-condition fold change (M) vs. mean expression (A) plots in the top row in Figure 5 show that the identification of differentially expressed genes is tightly and directly linked to their expression level for each tool. In order to be identified as differentially-expressed, transcript abundances must pass a threshold that is a composite of the mean expression level and the between-condition expression difference. This type of plot, while commonly used, obscures the relationship between within- and between-condition variation since the plasmid-associated genes circled in black are not obviously separated from the rest.

The bottom row of Figure 5 shows a plot of the between-condition fold change (M) vs. within-condition fold change (W) (MW). Here the majority of the plasmid-associated transcripts, circled in black, are clear outliers on the within-condition axis. Note that the within-condition transcript variation ranges from a value of approximately 2 to greater than 15. This is because the gene itself is present in one sample from each condition, but the transcript abundance varies greatly. Therefore, the expression difference for a given transcript is controlled both by gene presence and by transcript abundance. Transcripts from these genes should not rationally be identified as differentially expressed between-conditions. Both CuffDiff and edgeR identified approximately half of these transcripts as differentially expressed, but DESeq and ALDEx did not. However, all tools except ALDEx identified many differentially expressed transcripts where the within-condition variation was larger than the between-condition variation, with DESeq identifying the fewest.

The number of differentially-expressed transcripts identified by each method is shown in the Venn diagram in Figure 6. ALDEx identified the largest number of differentially-regulated genes in this dataset, but over 75% of these were also identified by the other 3 methods. Only 4.5% of the differentially-regulated genes identified by ALDEx were unique. One recent paper used microarrays to characterize the response of *B. subtillus* ATCC 14579 to a variety of acid responses, including a 30 minute acid downshock with HCl to pH∶5.0[40] at various times after the downshift. We extracted the list of genes that responded to HCl, but not organic acids from the supplementary tables as those that were up- or down-regulated in the following categories: responds to all acid downshocks in microarray, responds to HCl but not acetic or lactic, responds to nonlethal acid downshocks by any acid, and responds to HCl only with retarded growth. We examined the overlap between the four RNA-Seq analysis methods and the microarray analysis of acid response and found that all four methods were able to identify equivalent numbers of the 423 genes that responded to specifically to HCl in this experiment (number of differentially-regulated genes identified by method: edgeR 376, ALDEx 377, DESeq 383, CuffDiff 390). While the experimental design of the microarray is not an exact duplicate of our analysis, these results shows that all four methods, including ALDEx, are able to identify the genes that are differentially regulated during a known biological response.

**Figure 7:**
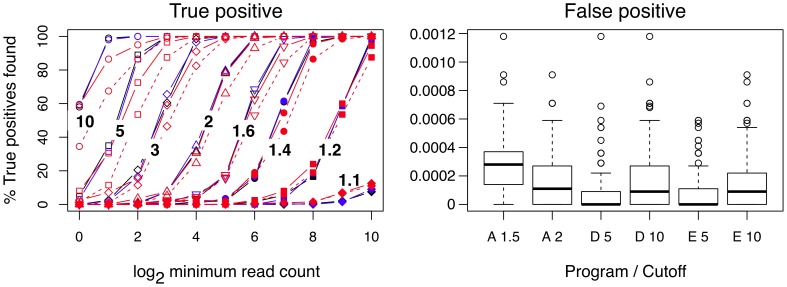
True and False positive identification in simulated data. A set of eleven genes with simulated read counts between 1 and 1024 in two-fold increments were appended twice to a single sample of the *B. cereus* dataset. Two conditions were generated by multiplying the counts for a single set of simulated genes in each condition by the fold-difference values indicated in the True positive panel on the left, and two simulated technical replicates were generated for each condition by sampling from the Dirichlet distribution which accurately models technical variance in these datasets (Figure 1). The resulting four samples were examined by DESeq, edgeR and ALDEx for the ability of each method to identify the simulated differentially-expressed genes. The fold change varied between 1.1 and 10 and 100 simulations were run for each fold change. The fold change value is overlaid on the corresponding curve in the left panel. The line colors are black for edgeR, blue for DESeq and red for ALDEx, and the symbols are the same for each fold change value across each method. The ALDEx 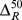 cutoff of 1.5 is a solid and 2.0 is a dashed line. The right panel shows the per-gene false positive rate for each method at two cutoffs. False positive events in this model can only arise through outliers in the Dirichlet sampling procedure. The rate was calculated by dividing the number of false positive genes identified in each trial by the number of genes in the dataset (5358). The boxplot shows the range of false positive rates observed for each method across all trials and all expression levels. A rate of 0.0002 corresponds to approximately 1 false positive per trial in this dataset.

In addition, we compared ALDEx to DESeq and edgeR using a synthetic dataset based on the *B. cereus* dataset. Here, an additional 22 synthetic genes were known to be differentially expressed by fold differences ranging between 1.1 and 10 with initial read counts ranging between 1 and 1024. Technical variance within and between the conditions was modelled by Dirichlet sampling. Figure 7 shows the results. As expected, we observed that the ability of DESeq and edgeR to identify true positive expression differences were nearly indistinguishable, as was their average per-gene false positive rate when examined at the same false discovery rate cutoff. When ALDEx was used with the 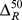 of 1.5, it performed nearly as well in these simulated datasets as did the other two, but was slightly less sensitive at low simulated gene expression levels. This result is entirely consistent with the underlying ALDEx algorithm, as the technical variance is large when expression levels are low (Figure 1). As expected ALDEx with a 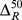 cutoff of 2.0 was even more restrictive at low expression levels, and was somewhat less sensitive than the other methods, although the difference is small when the minimum expression level was greater than 4 counts per gene. The right panel shows the per-gene false positive rate calculated for ALDEx at 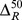 of 1.5 and 2.0, and for the other two methods at a FDR of 5% and 10%. All three methods were found to have low false positive rates. ALDEx with a 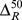 of 1.5 was the highest, although even here the per-gene false positive rate translates into approximately 1.5 false positive gene identifications in the 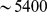 gene dataset. The ALDEx 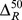 cutoff of 2.0 had a per-gene false positive rate that was essentially the same as a FDR of 10% for the DESeq and edgeR algorithms.

We next compared ALDEx, edgeR and DESeq on the highly variable Meta-RNA-Seq dataset. CuffDiff was not used as it was not practical to generate the required input gff files from the mixed-species sample. This dataset contained two clinical samples from vaginal swabs obtained from non-BV (bacterial vaginosis) women and one each from a women with intermediate- or full-grade BV[31]. The non-BV samples were composed largely of reads mapping to *Lactobacillus crispatus* and *Lactobacillus iners* and two BV samples contained reads from a more diverse population of organisms including the two *Lactobacillus* species, *Gardnerella vaginalis*, *Prevotella* species and others.

**Figure 8:**
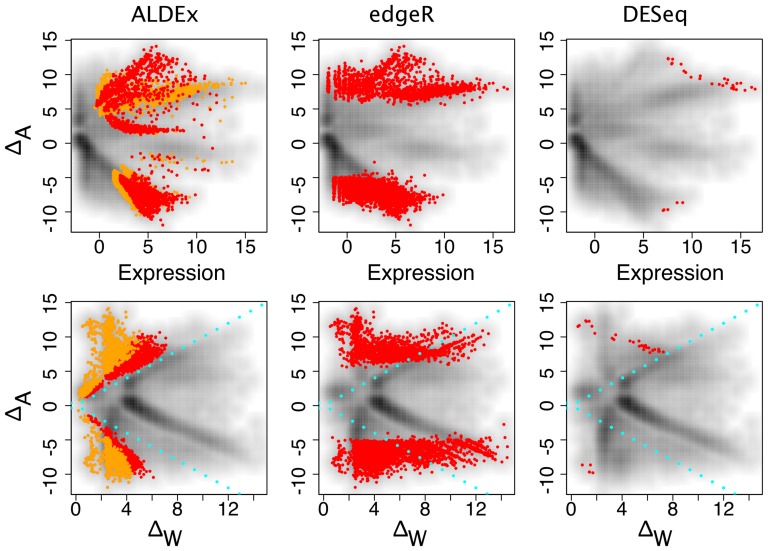
Comparison of three differential expression methods in the Meta dataset. This dataset contains extreme transcript abundance variation within- and between-conditions. In this dataset the ALDEx method exhibits similar characteristics as in the *B. cereus* dataset, in that it identifies as differential those transcripts that exhibit high between-condition variation and low within-condition variation. This is illustrated by the left column that shows an MA-like plot and an MW plot. It is obvious that the high levels of variation in the Meta dataset is a poor fit to the negative binomial model used by both edgeR and DESeq. The edgeR package appeared to enforce a relatively high between-condition differential expression level regardless of the mean expression value. This leads to many poorly expressed transcripts being identified as differentially expressed. In contrast, the DESeq package identified as differentially expressed a small number of highly expressed genes. As before, transcripts with differential abundances are coloured red (and orange) as per the rules outlined in Figure 5.

The three tools behaved very differently in this dataset as shown in Figure 8. ALDEx and edgeR identified a large number of transcripts as being differentially expressed, although it is apparent that there was minimal overlap between the transcripts identified. The MA and MW plots for ALDEx illustrate that this tool identified as differentially expressed those transcripts that met the following criteria: non-negligible expression, and within-condition variation lower than between-condition variation. In contrast, edgeR identified transcripts with large fold expression differences regardless of the average expression level or the within-condition variation. DESeq was very conservative and identified only a handful of differentially-expressed transcripts, all with extremely high mean expression levels.

**Figure 9:**
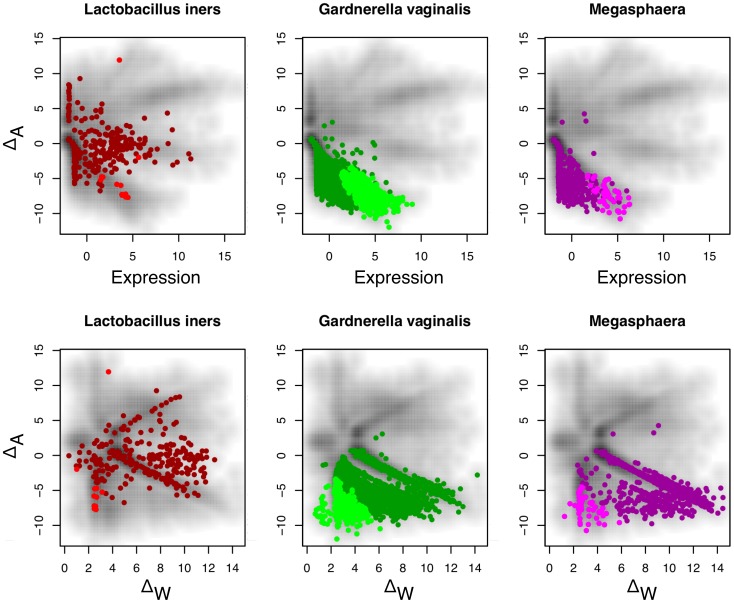
The effect of organism abundance on transcript abundance in the Meta dataset. In each panel, the dark colour indicates a gene that maps to that organism (or organism group) and the bright colour indicates a gene that is differentially expressed according to the rules enforced by ALDEx. The Meta dataset contains different mixtures of organisms in each sample as shown in Table 2. This leads to widely different distributions of transcript abundance in this dataset, which can be classified into three general patterns. Transcripts from organisms that are abundant (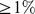) in three or four samples can exhibit both up and down regulation in the two conditions. For example, transcripts that were derived from *L. iners* are seen to be both up- and down-regulated in this dataset. Transcripts from organisms that are abundant in both samples of one condition (e.g. *G. vaginalis*), exhibit a change in one direction only, and show the full range of within-condition variation. In this case, many genes from the organism are expressed concordantly, and can be identified as differential. Transcripts from organisms that are abundant in only one sample of one condition, e.g. *Megasphaera* species, show a similar pattern as the previous one, but there is much more extreme variation within-conditions, and only those few genes that are expressed at extremely high levels can be reliably called as differentially expressed.

It is likely that both edgeR and DESeq performed poorly because the underlying assumptions of their statistical models was a poor fit for the data. In particular, the points in Figure 8 appear to have some underlying structure. This was explored by overlaying the organism-of-origin of each seed sequence for each clustered gene on top of these graphs, and highlighting the differentially-expressed transcripts identified by ALDEx. Three different patterns were observed and are shown in Figure 9. *L. iners* exemplifies the first pattern, where the organism is found in either 3 or 4 samples. Here, the transcript abundances and within- and between-condition differences are distributed widely throughout the plots. The second pattern is similar to that for *Gardnerella vaginalis* and is typical for organisms that are abundant in both samples of one condition and absent from both samples of the other condition. Here the transcripts are abundant in one condition only, exhibit a wide range of expression values and tend to the lower end of the within-group difference. The final pattern is similar to that for *Megasphera*, which was abundant in only one sample of one condition, and rare or absent in the others. Here the average expression values tend to the lower average expression range and the within-condition difference is at the upper range of values. Note the difference between the number of differential transcripts for *Gardnerella* vs. for *Megasphaera* which reflects the typically lower within-condition transcript variation. Taken together, these plots show that the expression levels of a transcript in a Meta-RNA-Seq experiment, and the variation of those levels within- and between-conditions are driven by two factors. The first, is the abundance of an organism across samples, and the second is the transcript abundance within an organism. Transcripts derived from organisms that are found in all samples form a subset that have distributions similar to those for single-organism RNA-Seq, and therefore *in isolation* might be amenable to analysis with existing tools. However, transcripts derived from organisms that are found in only one condition, or only one sample, clearly deviate from this ideal and identifying differential transcripts using ALDEx provides an approach that can find those genes that are consistently different between conditions benefits from this approach.

## Discussion

While the cost of high-throughput sequencing continues to fall, conducting an RNA-Seq experiment is still a relatively expensive undertaking. Extracting and analyzing the results adds an under-appreciated layer of complexity and cost[41]. The analysis of single-organism RNA-Seq has largely been informative with fixed-effect models, although the recent versions of both DESeq and edgeR have incorporated statistical methods to better deal with intra-condition variation[23], [38], [42]. However, as shown here and discussed elsewhere[43] there are acknowledged challenges that are magnified in the examination of Meta-RNA-Seq datasets.

One current approach to examining metatranscriptomics has been championed by the marine metagenomics community that uses pyrosequencing and a non-parametric bootstrap test[44] to evaluate differences in gene content and gene expression. However, Parks and Beiko[13] recently demonstrated that the level of significance achieved by this method is sensitive to the number of bootstrap samples. Drawing more samples from the pooled dataset results in smaller 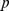 values and in more features being identified as significant. Sensitivity to the number of bootstrap samples is indicative of there not being enough data for the bootstrap procedure itself to be valid[45]. Aside from numerical convergence, 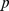-values cannot be interpreted as effect-sizes. Thus while non-parametric bootstrapping has been widely adopted in the marine metagenomics community (where a defined protocol is followed [20], [46]-[48]) it is unlikely to be adopted for use by the wider community.

Others have used the Illumina platform to generate Meta-RNA-Seq data composed of millions of reads and have used more diverse statistical tools drawn from the metagenomic armamentarium that are largely concerned with identifying gene presence or absence as opposed to gene abundance over a large range. Statistical analyses in these studies have ranged from simple, such as listing those genes with 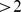-fold changes[21], to sophisticated, such as the adaptation of the Metastats differential 16S rRNA abundance tool for RNA-Seq[49] or the application of the well-known meta-genomic program, ShotgunFunctionalizeR[50], [51]. Note that the ShotgunFunctionalizeR program[52] uses a Poisson-based model to characterize the differences in gene count between meta-genomic samples, and so is expected to have similar properties as DESeq or edgeR with highly dispersed data.

To our knowledge, ALDEx is the only method capable of producing an estimate of the ratio of between-condition difference to variability seen within the different conditions for RNA-Seq data since it assumes a random-effects model. We examined the behaviour of two widely-used tools based on fixed-effect models: edgeR and DESeq, both based on negative binomial models. It is important to note that, in essence, both tools effectively estimate *two* parameters from *one* value by assuming that the data follows idealized behaviour. This approach works acceptably in datasets with high intra-condition reproducibility, but is prone to error if the dataset deviates from that ideal, as is likely true for the majority of Meta-RNA-Seq data. For example, the *B. cereus* dataset, while having an extremely high read density, does not display a high concordance with either of these fixed-effect-model methods. As outlined below, this is caused, in part, by the failure of existing tools to centre the data in such a way that proportionality is preserved.

ALDEx uses a Dirichlet-multinomial model to infer transcript-abundance from read counts, followed by mixture-modelling to explicitly account for within- and between-condition variation among experimental samples. These data are examined in an ANOVA-like framework with conservative cutoff values that identify differentially-expressed genes where the within-condition expression variance is much smaller than the between-condition variance. A further innovation is that the data is transformed by removing the scaling-dependent subspace inherent when proportional data is log-transformed, ultimately removing a potential large source of multivariate statistical bias (see Supplementary Figure S1 in File S1). This simple manipulation sets the gene expression coordinates of the experiment to the origin and removes the bias inherent in the log-transform; the across-gene mean log-expression value for a given sample is zero, and the mean expression change between-conditions is also zero (as is typical of ANOVA designs). One important feature of this transform is that it removes the necessity for an empirical LOWESS correction. Furthermore, the transform also converts all the relative-expression values to points in a Euclidean space that can then be added, scaled, or grouped in a manner that, while perhaps counterintuitive, are entirely consistent with the intuitive properties of objects for which the ''total amount'' is irrelevant [17].

We use both relative fold difference (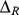), a measure of the effect size, and the distribution of absolute fold differences (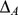) to identify differentially expressed genes. The procedure ensures that no gene is called as differentially expressed if the within-condition variation is less than some multiple of the between-condition variation. This makes intuitive statistical sense because we have weak evidence for differential expression if the within-condition variation is large. We have attempted to design and describe ALDEx in a manner that formally captures an intuitive procedure that helps answer the question ''is this gene differentially expressed between conditions'' when (a) sample sizes are prohibitively small, (b) read counts are relatively low thus implying that point-estimates of expression intensities will be imprecise, and (c) we do *not* wish to make assumptions about variance-sharing, preferring instead techniques more in line with robust estimation methods. ALDEx therefore attempts to robustly address the question ''is the observed between-group difference somehow `substantially bigger' than the within-group difference?'', a statement suggestive of classical ANOVA. However, rather than assuming normality, then assuming that a 5% FDR implies cutoffs of 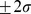, we simply say ''if the between-group difference is greater than four times the within-group difference'', corresponding to 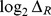 cutoffs of 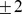, then that gene is of interest.

At least one other RNA-Seq analysis method, baySeq[53], has used a Bayesian framework to estimate the likelihood of differential gene expression. There are several differences between the Bayesian methods implemented in ALDEx and those implemented in baySeq. Firstly, baySeq estimates the posterior likelihood of differential gene expression in the context of a negative binomial model. Secondly, baySeq assumes that genes with 0 reads are not expressed, while ALDEx uses the more general assumption that genes with 0 reads are either not expressed or are expressed below the threshold of detection. Thirdly, ALDEx normalizes the estimated proportion vector using the isometric log transformation[18] while baySeq and all other existing methods do not. It is worth noting that a recent comparison of baySeq, DESeq and edgeR using a deeply-sequenced and validated dataset showed that DESeq and edgeR were more discordant with each other than either was with baySeq[54], suggesting that baySeq exhibits a lower false positive rate than either of the other methods. Finally, baySeq can deal with more complex study designs than can ALDEx.

In Meta-RNA-Seq datasets, i.e, multiple-organism, multiple-condition datasets, existing methods based on idealized behaviour are prone to both over- and under-calling differentially-expressed genes especially if within-condition variance is not explicitly accounted for. However, ALDEx maintains the logical consistency of only identifying genes with low within-condition dispersion and high between-condition differences. This approach has been used successfully to examine differences in gene expression of a vaginal community in two states[31]. The approach should be applicable to other situations where high-throughput sequencing is used such as metagenomic analysis of differential gene abundance and ChIP-Seq.

## Materials and Methods

*Bacillus cereus* 14579 and an isogenic derivative that carries a deletion of a chromosomal encoded minor sigma factor gene and has lost the pBClin15 plasmid were each grown at 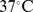 with aeration in LB medium buffered with 10 mM MES and 10 mM MOPS. When the culture reached an optical density (OD600) of 0.5 a sample was taken (pH∶7.2) before addition of 1 N HCl to shift the culture to pH∶5.46. After incubation for 20 minutes the low pH sample was taken. Both samples were processed identically by immediately adding the aliquot to an equal volume of acid-phenol:chloroform (5∶1) pH∶4.5 (Ambion) at 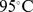. After 5 minutes with periodic mixing the aqueous and organic layers were resolved by centrifugation. The aqueous layer was further extracted at 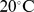 with 1 volume of phenol:chloroform:isoamyl alcohol (25∶24∶1) pH∶6.6 (Ambion). RNA was recovered from the aqueous phase by precipitation with isopropanol and then dissolved in TE buffer (10 mM Tris-HCl, pH∶7.5, 1 mM EDTA) buffer. Residual DNA was removed by treatment with TURBO-DNase (Ambion) followed by purification of the RNA on a RNeasy mini-column (Qiagen). Ribosomal RNA was subsequently depleted with the MICROBExpress procedure (Ambion).

### Ethics Statement

The Health Sciences Research Ethics Board at the University of Western Ontario granted ethical approval for the study under approval number REB16183E. Participants gave their signed informed consent before the start of the study. Premenopausal women between the ages of 18-40 years were recruited at the Victoria Family Medical Center in London, Canada. Participants were excluded from the study if they reached menopause, were menstruating, had a urogenital infection other than BV in the past 6 months, were pregnant, had a history of gonorrhoea, chlamydia, estrogen-dependent neoplasia, abnormal renal function or pyelonephritis, were taking prednisone, immunosuppresives or antimicrobial medication, had undiagnosed abnormal vaginal bleeding, had engaged in oral or vaginal intercourse or consumed probiotic ements or foods in the 48 hours prior to the clinical visit.

### Clinical Samples and RNA preparation

Vaginal swabs were collected from four women: two with BV and two considered to have a non-BV vaginal biota as diagnosed by Nugent scoring[55], and vaginal pH. Nurses obtained vaginal samples for RNA-seq using a Dacron polyester-tipped swab rolled against the mid-vaginal wall and immediately suspended in RNAprotect (Qiagen) containing 100 µg/ml rifampicin. Vaginal pH was measured using the pHem-alert applicator (Gynex). Samples for RNA extraction were incubated at room temperature for at least 10 minutes (to a maximum of 3 hours), and then centrifuged before discarding the supernatant and freezing the remaining pellet at 80

C. Lysis and RNA extraction were performed within 3 weeks of storage. RNA was isolated as for the *B. cereus* samples.

Reference sequence clustering and mapping. A total of 110 accessions representing 103 organisms (of 31 genera, and 63 species) isolated from or detected in the vagina were included in a reference sequence set for mapping. These 234,991 sequences (including 230,031 coding sequences) were clustered by sequence identity (95% nucleotide identity over 90% sequence length) using CD-HIT[56] to remove redundancy in the reference mapping set. A representative sequence (''refseq'') from each of the resulting 163,014 clusters was used to build a Bowtie[32] colorspace reference library for mapping the RNA-seq reads. Reads mapped uniquely by Bowtie to a coding refseq were included in the differential expression analysis (all other unmapped reads were discarded). Reads were trimmed from the 3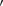 end to 40 nt, and up to 2 mismatches were allowed.

ALDEX version 1.0.3 was used. It can be accessed at: http://code.google.com/p/aldex/. DESeq version 1.6.1 was used for these analyses using the per-gene dispersion estimates. The edgeR version 2.4.6 package was used. A false discovery rate of 0.1 was used to identify putative differentially-expressed transcripts as recommended by the documentation. Cuffdiff version 1.3.0 was used with a mean fragment length of 200 bp and the default false discovery rate of 0.05.

## Supporting Information

File S1 Supporting information.
(ZIP)
