# Supplementary material for: ANOVA-Like Differential Expression (ALDEx) Analysis for Mixed Population RNA-Seq
Source: PLoS One. 2013 Jul 2;8(7):e67019. doi: 10.1371/journal.pone.0067019 (PMC3699591; doi:10.1371/journal.pone.0067019)
Supplement: File S1 — Supporting information. (ZIP) [file pone.0067019.s001.zip › supplementary_info_final.pdf]

Supplementary information for ANOVA-Like Differential Expression (ALDEx) analysis for mixed population RNA-Seq

Andrew D. Fernandes, Jean M. Macklaim, Thomas G. Linn,

Gregor Reid and Gregory B. Gloor

October 18, 2012

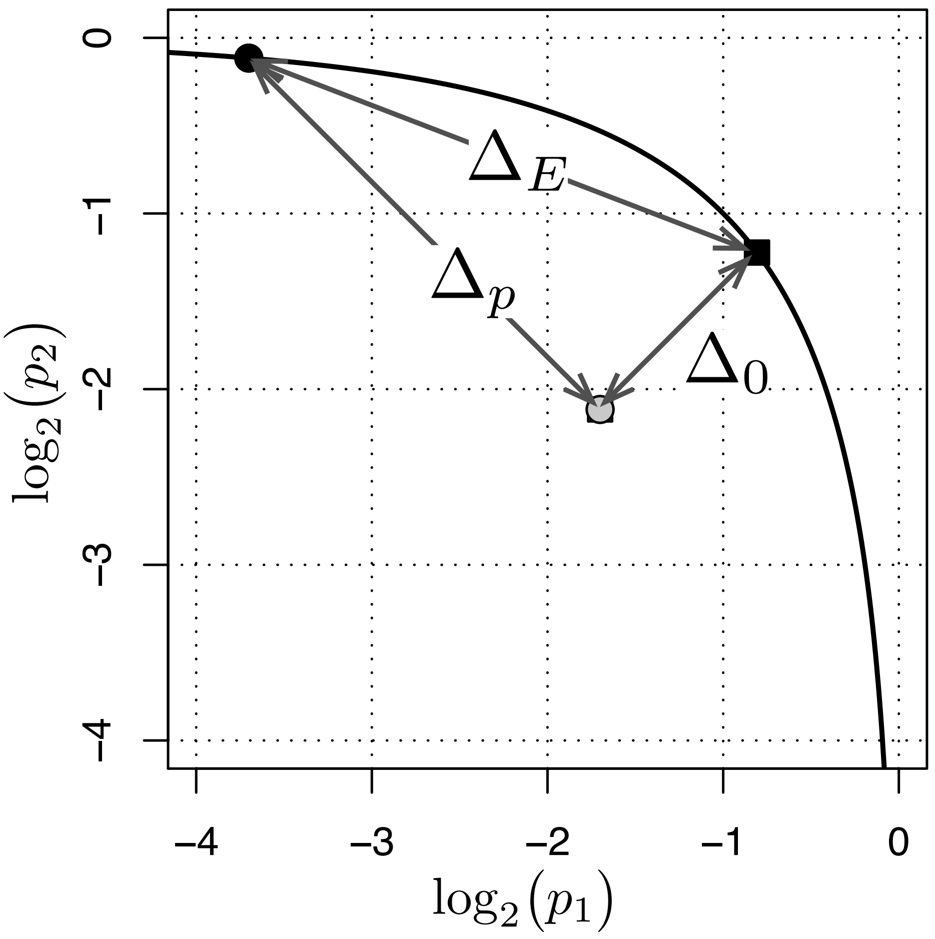

Figure S1: **Removing the bias of the log-transformation.**

Consider a two-gene experiment where the proportional expression is represented through p_1 and p_2 where $p_1 + p_2 = 1$. When log-transformed, the linear constraint $p_1 + p_2 = 1$ becomes the solid black curve $e^{\log(p_1)} + e^{\log(p_2)} = 1$. Consider the problem of determining the differential expression level between two samples, one represented by (●) and the other by (■). Simple subtraction yields the differential-expression vector Δ_E. However, since adding a constant to each of the pair $[\log(p_1), \log(p_2)]$ does not change their relative (non-log) proportion, we subtract the vector Δ_0 from $[\log(p_1), \log(p_2)]$ leaving the *true* difference Δ_p. The component along the Δ_0 line (subspace) is systematic bias introduced by the non-linear log-transformation, and is parallel to the line between $[0, 0]$ and $[1, 1]$.

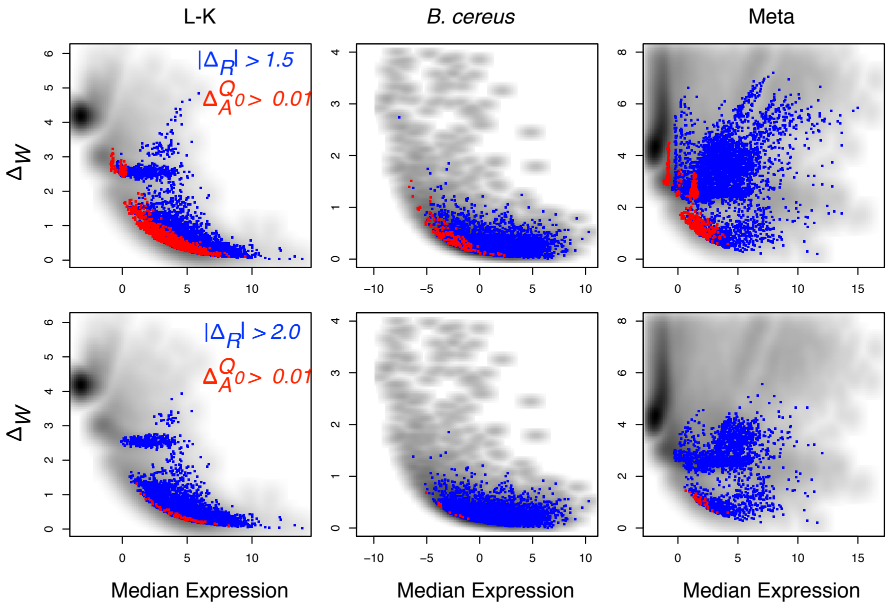

Figure S2: **The $\Delta_A^{Q_0}$ cutoff preferentially excludes genes with low expression.**

The plot shows the within-condition gene-expression difference to the median expression level for three different experiments. Blue points identify genes that pass the $|\Delta_R| >= 1.5$ (top) or $|\Delta_R| >= 2.0$ (bottom) cutoff values. Overplotted in red are genes that pass this initial test, but are excluded because they have $\Delta_A^{Q_0}$ values ≥ 0.01. Note that for any given Δ_W value, the genes excluded by the $\Delta_A^{Q_0}$ test are those with the lowest mean expression value. This general observation holds even for the conservative cutoff of $|\Delta_R| >= 2.0$. Also note that the red is overlaid on the blue, and the apparent number of excluded genes is greater than the actual number.
